# Docosahexaenoic Acid Inhibits Vascular Smooth Muscle Cell Proliferation Induced by Glucose Variability

**DOI:** 10.2174/1874091X01711010056

**Published:** 2017-06-30

**Authors:** Kaliyaperumal Rani, Nway Y. Aung

**Affiliations:** Nanyang Technological University Singapore, Singapore

**Keywords:** Apoptosis, Diabetic atherosclerosis, Docosahexaenoic acid, Intermittent high glucose, Proliferation, Stable high glucose, Vascular smooth muscle cells

## Abstract

**Background:**

Vascular Smooth Muscle cells (VSMC) enact crucial roles in early vasculogenesis and sustenance of vascular integrity. However, aberrant proliferation of VSMC followed by migration into the blood vessel wall leads to the formation of vascular lesions accounting for the degeneration and remodelling of vascular basement membrane. In diabetes, hyperglycaemia accelerates VSMC proliferation and contributes to the initiation and progression of atherosclerotic lesions. Recently, acute glucose fluctuations have been implicated in the abnormal VSMC proliferation and complications of diabetic atherosclerosis. Docosahexaenoic acid (DHA), a ω-3 polyunsaturated fatty acid (PUFA) has been shown to inhibit proliferation of several cell types implicating several different mechanisms. In the present study, we have investigated the effects of DHA on VSMC proliferation induced by stable and intermittent high glucose levels.

**Method:**

Confluent cultures of rat aortic VSMCs were treated with DHA for 24 hrs and then exposed to stable high glucose (25 mmol/L, SHG) or intermittent high glucose (5 mmol/L and 25 mmol/L alternating every 12 hrs, IHG) for 72 hrs. Cell proliferation was examined by the MTT viability assay, while apoptosis process was evaluated by the Hoechst staining, flow cytometry and caspase-3 activity assays.

**Results:**

Our data demonstrated that the hyper proliferation induced by stable and intermittent high glucose levels was significantly inhibited by the DHA pre-treatment. DHA significantly increased caspase-3 activity, resulting in enhanced DNA fragmentation and apoptosis.

**Conclusion:**

Our results suggest that DHA reduced the high glucose-induced proliferation of VSMC and induced cell apoptosis.

## 1 INTRODUCTION

Atheroscelerosis-associated macrovascular problems such as myocardial infarction and cerebrovascular diseases contribute to the major causes of morbidity and fatality in diabetic populations [1]. Hyperglycaemia hastens the progression of atherosclerotic lesions by elevating the atherogenic potency of vascular smooth muscle cells (VSMCs) in diabetes patients [2]. Aberrant VSMC proliferation along with its migration into the blood vessel wall leads to the formation of vascular lesions accounting for the degeneration and remodelling of vascular basement membrane [3, 4]. It has been hypothesized that an imbalance between exaggerated proliferation and depressed apoptosis might be involved in abnormalities of VSMC growth associated with cardiac complications [5]. Therefore, restoring the delicate balance between VSMC proliferation and apoptosis may offer effective treatment strategy for diabetic atherosclerosis.

Chronic hyperglycaemia has been recognised as the primary causal factor for macrovascular complications preceding to accelerated atherosclerosis in diabetes. Both fasting and post prandial plasma sugar levels account for this process. Recently, acute glucose fluctuation in diabetes is identified as an additional factor that triggers the diabetes complications [6]. Hence, “hyperglycaemic vacillations” might be entitled with unambiguous and pivotal responsibility in the pathogenic diabetic vascular complications [7]. For instance, in human umbilical vein endothelial cells (HUVECs), intermittent high glucose (IHG) has been evinced to stimulate the overproduction of reactive oxygen species (ROS) and to contribute towards enhanced cellular apoptosis [8]. Previous studies indicated that IHG accelerated the number of malfunctional β cells provoking apoptosis, a condition known as β cell glucose toxicity [9, 10]. IHG induced a higher manifestation of adhesive molecules such as ICAM-1, VCAM-1 and E-selectin in HUVECs than that of stable high glucose (SHG) [11]. A recent study on VSMCs found that IHG accelerated cell proliferation and is facilitated by enhanced osteopondin (OPN) synthesis, a cell adhesion molecule that can bind with the VSMCs surface integrin receptor, and mediate cellular adhesion, proliferation and migration [6]. These findings demonstrate that the inconstancies of glucose level contribute directly to the vascular complications in diabetic population.

Salutary effects of foods, enriched with marine-derived ω-3 polyunsaturated fatty acids (PUFA), in treating cardiovascular diseases are widely accepted. Epidemiology results also demonstrate that regular consumption of ω–3 PUFAs helps in minimizing the fatality rate of cardiovascular diseases [12, 13]. In particular, a decline trend exhibited in atherosclerosis inflammatory pathology and the decreasing heart attack fatality rate of diabetes patients were associated with increased ω-3 PUFA intake [14-16]. The defense mechanism through which ω–3 PUFA functions remains obscure but are anticipated to be ascribable to their actions on endothelial and vascular smooth muscle cell functions [17]. For instance, docosahexaenoic acid (DHA), a ω-3 PUFA, was found imperative in alternating the signal transduction mechanisms in various cell types [18, 19]. DHA has also been identified in conducting anti-tumorigenic pathway correlated with inhibition of tumor-mediated angiogenesis [20, 21]. In human endothelial cells, DHA was reported to protect the cells from stress-induced apoptosis [22]. In VSMCs, DHA influences vascular remodeling and restenosis [23]. In the present study, we investigated the effects of DHA against the exhilarated proliferation of rat VSMCs exposed to SHG or IHG in culture.

## 2 MATERIALS AND METHOD

### 2.1 Materials

Dulbecco’s modified Eagle’s medium (DMEM), penicillin, streptomycin, trypsin, dimethyl sulfoxide (DMSO) and foetal bovine serum were purchased from Gibco, Singapore. 3-(4, 5-dimethylthialzal-z-yl)-2,5-diphenylterazolium bromide (MTT), Hoechst 33258 dye and DHA (22:6n-3) were procured from Sigma-Aldrich (Singapore). Caspase-3 Fluorescence Assay kit was from Cayman chemicals (Michigan, USA). The other reagents were all from Sigma Chemical Co (Singapore). DHA was dissolved in 99.5% ethanol and stocked as aliquots at -80˚C. As DHA is highly susceptible to oxidation, every aliquot was used not more than two times.

### 2.2 Cell Culture

Rat aortic VSMC purchased from American Type Culture Collection (ATCC, Manassas, USA) was used for this study. Cells were cultured in DMEM consisting of 5.5 mmol/L glucose, 10% fetal bovine serum, 100 U/ml penicillin and 100 U/ml streptomycin at 37˚C (humidified atmosphere with 5% CO2). Cells at passages 7-9 were used for the experiments.

### 2.3 Experimental Procedures

VSMCs were trypsinized into cell suspension and seeded in each well of 96-well plate with density of 10^4^ cells per well. When the cells became confluence (70-80%), serum-free DMEM was used to replace the medium and cells were allowed to synchronize for 24 hrs. Synchronized cells were then split into eight different categories: normal control group (Con), SHG group, IHG group, DHA control group (DHA-Con), DHA-SHG group, DHA-IHG group and osmotic control groups (OS1 and OS2). In the Con group, cells were exposed to normal concentration of glucose (5.5 mmol/L). The cells in SHG group were treated with stable high concentration of glucose (25 mmol/L) whereas those in IHG group, they were treated with intermittent concentrations (fluctuating between 5.5 mmol/L and 25 mmol/L within 12 hrs) and were incubated for 72 hrs. In DHA-SHG group, the cells were pretreated with 20 µM DHA and exposed to stable glucose whereas in DHA-IHG group, the cells were pretreated with 20 µM DHA and exposed to intermittent concentrations of glucose for indicated period. The cells in DHA-Con group were incubated with identical amount of DHA without any further treatment. Among the two osmotic controls, one was treated with mannitol (44.4 mM) plus normal glucose (5.5 mmol/L) as a common high osmotic control; the other represents an intermittent high osmotic control (alternating between 5.5 mmol/L glucose or mixture of mannitol and 5.5 mmol/L glucose every 12 hrs). Equal quantities of ethanol were used to incubate the control groups and the concentration in the medium was maintained at less than 0.1%. Cells in all different categories were treated under identical conditions and all experiments were conducted three times at least.

### 2.4 Cell Proliferation Assay

Cell proliferation was evaluated by estimating the metabolism of 3-(4, 5-dimethyldiazol-2-yl)-2, 5-diphenyltetrazolium bromide (MTT). In brief, VSMCs seeded in 96-well microtiter plates were pretreated with 20 µM DHA and exposed to either constant or intermittent high glucose for 72 hrs. After the treatment, 20 *μ*L of MTT solution (5 mg/mL) was mixed in all the wells and cells were incubated at 37°C for another 4 hrs. The solution was disposed of and 150 *μ*L of solubilisation solution (DMSO) was added into each well followed by 10 mins shaking. Absorbance of the solubilised blue formazin dye was read at 570 nm using a microplate reader. Cell viability was represented as a percentage of cytoprotection versus control group, fixed at 100%.

### 2.5 Morphological Analysis by Hoechst 33258

For morphological evaluation of nuclei, VSMCs were stained using nuclear dye Hoechst 33258. Hoechst 33258 fluorochromes have been used to define the nuclear morphological features that act as a quantitative index of apoptosis in vast majority of cell culture systems [24, 25]. After treatment, cells were stained with Hoechst 33258 (10 *μ*g/mL) at room temperature in the dark for 30 mins and washed three times with PBS. Finally, cells were illuminated with ultraviolet light and nuclear morphological transformations, such as condensed and disintegrated nuclei with intense bright fluorescence, were visualized under fluorescence microscopy.

### 2.6 Apoptosis Analysis by Flow Cytometry

Apoptosis was estimated quantitatively through the analysis of propidium iodide (PI) staining in flow cytometry. In brief, VSMC grown in 25 cm^2^ culture flasks, after being treated with glucose, were harvested by trypsin and washed with PBS. Cells were then fixed with 70% ice cold ethanol at 4°C overnight. Next day, cells were washed twice with cold PBS followed by incubation with 100 µg/ml RNase at 37°C for 20 mins. After that, cells were centrifuged (10 min, 1800 rpm at room temperature) and the pellets were subjected to incubation with PBS containing 50 µg/ml PI at 37°C in the dark for 30 mins. Samples were analysed using flow cytometer (Becton Dickinson FACScan) and flowing software.

### 2.7 Caspase Activity Assay

Caspase 3 activity was assessed through monitoring the fluorescence of t N-Ac-DEVD-N’-MC-R110 substrate with respect to the manufacturer’s instructions (Cayman Chemical, USA). VSMCs seeded in 96-well plate were treated as described above, and the cell lysates were prepared by adding 100 µl cell lysis buffer (Cayman Chemical) followed by shaking for 30 min at room temperature. After centrifugation at 800 x g for 10 min, 100 µl of supernatant was shifted to another plate followed by incubation at 37°C with 100 µl of N-Ac-DEVD-N’-MC-R110, a caspase 3 substrate. Fluorescence of the cleaved substrate was measured at an excitation wavelength at 485 nm and an emission wavelength at 535 nm using a microplate reader.

### 2.8 Data Analysis and Statistics

Paired student’s t-test was used to analyse the data obtained. Discrepancies of p values less than 0.05 were taken as statistically significant and the results were expressed as mean ± standard deviation (SD) attained from at least three experiments.

## 3 RESULTS

### 3.1 Effect of DHA on Proliferation of VSMCS Induced by IHG

It is known that IHG is more detrimental than SHG in hastening abnormal proliferation of VSMCs in diabetes [6, 26]. To test whether DHA modifies IHG-induced proliferation of VSMCs, quiescent cultures were pretreated with DHA and exposed to constant or intermittent high glucose concentrations for 72 hrs. After treatment, cell viability was measured using the MTT reduction assay.

As depicted in Fig. (**1**), cell viability of the Con group was fixed at 100%. The cell viability of VSMCs exposed to SHG was significantly increased to 160.35 ±1.88% (p*<*0.001) of the Con value. Furthermore, IHG was found to induce significant increase in cell viability more than SHG (192.25±1.71%, p*<*0.001). Pre-treatment of cells with DHA reduced the cell viability to 107.31±4.21% (p<0.001) in DHA-SHG group and to 108.93±6.74% (p<0.001) in DHA-IHG treated group. However, the Con groups pre-treated with DHA did not show any significant difference in cell viability. Also, there was no substantial discrepancy in the cell viability noted among the mannitol-treated and control group. These results indicated that the VSMCs exposed to IHG showed increased cell proliferation than SHG and pre-treatment with DHA significantly attenuated the induced proliferation in both SHG and IHG group.

### 3.2 Effects of DHA on Nuclear Morphology of VSMCS Exposed to IHG

Cell proliferation and apoptosis represent two different cellular events, however, they are often found intimately connected under physiological conditions. As reported by previous studies, any factor which restrains the viability of cell could suscitate the apoptosis of that cell or vice versa [27]. The effects of DHA on apoptotic index of VSMCs treated with HG was assessed by monitoring apoptotic morphological differentiations using bis-benzimide (Hoechst 33258) staining under a fluorescent microscope.

As illustrated in Fig. (**2**), the nuclei of Con cells contained evenly dispersed chromatin with sparse fluorescence signal showing almost no signs of morphological nuclear damage. Cells exposed to SHG and IHG also exhibited no chromatin disintegration or margination. In marked contrast, DHA treatment in SHG and IHG group demonstrated enhanced DNA fragmentation and condensation. However, treatment with mannitol did not instigate the apoptosis of VSMCs in both control groups.

The above results revealed that DHA treatment could result in increase of apoptotic cells in both SHG and IHG groups, further affirming that DHA induced cell death through apoptosis.

### 3.3 DHA Pre-treatment Induces Apoptosis of VSMCS Exposed to IHG Measured by Flow Cytometry

**Fig. (3):**
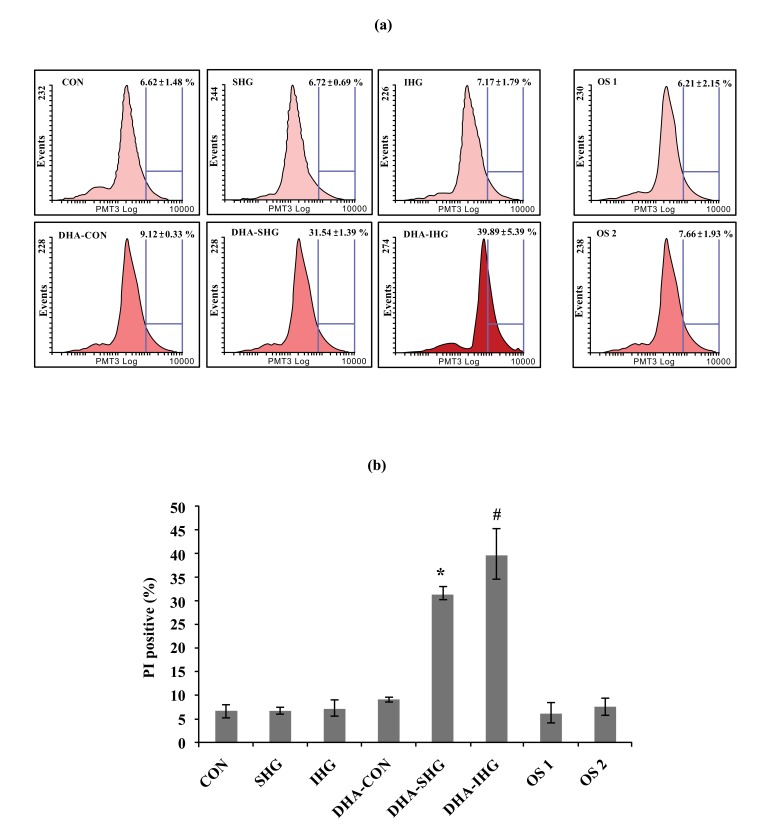
Effects of DHA pretreatment on apoptosis of VSMCs characterised by flow cytometry. (**a**) VSMCs were pretreated with DHA for 24 hrs and exposed to stable or intermittent high glucose for 72 hrs as mentioned in the text. The apoptosis rate of VSMCs was computed by FACS assay using PI staining. Results from 10,000 events were analyzed in each sample. (**b**) Quantitative analysis of apoptosis. Data were expressed as mean ± SD from three experiments. The percentages of apoptotic cells in DHA-SHG and DHA-IHG were increased significantly. * *P* < 0.001 versus SHG, # *P* < 0.001 versus IHG.

To quantitatively gain insight into the effect of DHA on apoptosis of VSMCs exposed to IHG, the apoptosis rate of VSMCs was computed by FACS assay using PI staining (Fig. **3a**). As shown in Fig. (**3b**), Con group revealed lower apoptosis rate of 6.62 ± 1.48%. Similarly SHG and IHG groups also showed reduced percentage of apoptotic cells. On the contrary, DHA treatment in SHG and IHG group triggered significant elevations of apoptosis with the apoptosis rate of 31.54 ± 1.39% and 39.89 ± 5.39% respectively. However, mannitol treatment did not show any changes in both control groups.

### 3.4 DHA Pre-Treatment Induces Caspase-3 Activity in IHG Treated VSMC

Cell apoptotic events, triggered by a diverse array of stimuli, focalize on the stimulation of caspase family, premier among which is caspase-3. Caspase-3 enacts an inevitable role in turning on the apoptotic processes in a wide range of cell types including smooth muscle cells [28]. In order to investigate whether DHA mediates its apoptotic effects through caspase-3, caspase-3 activity in VSMC was assessed after being exposed to SHG and IHG. The results revealed that high glucose treatment in both cases did not affect the basal caspase-3 activity when compared to the control Fig. (**4**). Besides, mannitol treatment in both osmotic control groups did not influence the basal caspase-3 activity. However, addition of DHA resulted in a 2.95-fold raise in caspase-3 activity in the SHG group (*P*<0.001) and a 3.65-fold increase in the IHG group (p<0.001), while no significant effects in the Con group could be shown.

## 4 DISCUSSION

Herein, we reported a novel finding that DHA inhibited the proliferation of rat aortic VSMCs induced by SHG and IHG. Persistent hyperglycaemia has been shown to stimulate the proliferation of VSMCs and to contribute towards the advancement of diabetic atherosclerosis [29-32]. Recent evidences demonstrated that intermittent hyperglycaemia also executes its influence on expedition of diabetic atherosclerosis supporting a pathophysiologic relationship between IHG and enhanced cardiovascular susceptibility in diabetes [33-35]. Also, it has been hypothesised that variability in glycaemic control might be even destructive to the VSMCs as compared to stable high glucose concentration [6]. Therefore, intermittent hyperglycaemia may act as an additional causative factor in the development of diabetic atherosclerotic complications. New therapies targeting acute glucose fluctuations thus represent a potential therapeutic approach to prevent or improve these conditions.

Previous studies investigated the potency of ω-3 and ω-6 PUFAs in suppressing tumour progression and demonstrated that DHA potently suppresses cancer cell growth, suggesting the influence of ω-3 PUFA in modulating cancer progression [36, 37]. DHA is also shown to increase apoptotic cell death in normal rat colonic cells [38, 39]. In proliferating human endothelial cells, DHA has been demonstrated to exert anti-angiogenic effects by inducing apoptosis [40]. In the present study, the inhibitory effect of DHA on HG-influenced proliferation of VSMC was established by MTT assay which demonstrated that DHA could inhibit VSMCs proliferation induced by HG and IHG.

Apoptosis enacts an inevitable role in both normal physiology and pathology in a variety of tissues [41]. Chromatin condensation is one of the prominent hallmarks of apoptotic cell death, which is brought about by activated caspases. In the present study, the results of Hoechst 33258 staining demonstrated that DHA treatment on VSMCs exposed to SHG or IHG could induce DNA fragmentation and nuclear chromatin hypercondensation. Also, flow cytometric analysis of PI staining provided the quantitative index of apoptosis induced by DHA-pretreatment in cells exposed to SHG and IHG. To further investigate the apoptosis pathway, caspase-3 activity was evaluated. Apoptotic process is activated via two main mechanisms; one involving death receptors and another one involving stress mediated activation of caspase-9. Eventually these two mechanisms synchronize to elicit caspase-3 activation. Hence, caspase-3 (also referred as CPP32/Yama) is considered as the primary executioner in the initiation of apoptosis [42]. In the present study, DHA pre-treatment significantly induced caspase-3 activity in VSMCs which illustrates an apoptotic effect of DHA in this cell system. Elevated caspase-3 activity along with an upsurge of apoptotic cells revealed that caspase-3 induce apoptosis associated signalling cascade with genetic expression resulting in the development of apoptosis. The control cells treated with DHA did not show any obvious signs of apoptosis and elevated caspase-3 activity, which suggests that DHA induced caspase-3 activity and apoptosis predominantly in HG-induced hyper proliferating cultures of VSMCs.

The molecular mechanism through which DHA may induce apoptosis in smooth muscle cells is poorly understood. Previous studies indicated that DHA activated apoptosis through disruption of mitochondrial transmembrane potential, transformation of plasma membrane phosphatidyl serine, escalated bax expression and activation of caspase-3 [43]. Our findings revealed that DHA repressed the stimulatory effects of HG and IHG on the proliferation of cultured VSMCs by inducing apoptosis. Also, the present data suggested that the inhibitory effect of DHA on HG induced VSMC proliferation was at least partially mediated by caspase 3. However, the current study did not fully investigate the molecular mechanism behind the apoptotic effect of DHA on VSMCs under HG conditions, and also the possible involvement of other caspases, which we plan to investigate in the future.

## CONCLUSION

In summary, it was found that pre-treatment of VSMCs with DHA inhibits the proliferation of VSMCs triggered by HG level. In addition to the stable high glucose, protective effects of DHA were also pronounced in cells exposed to IHG, indicating the protective role of DHA against vacillating hyperglycaemic states of diabetes. Results obtained in the present investigation indicate that apoptosis is most likely to be the essential mechanism through which DHA intercedes its anti-proliferative effects in VSMCs under HG conditions. Distinctive features of apoptosis such as chromatin condensation and nuclear fragmentation were clearly apparent in DHA-treated HG cells shown by the fluorescent microscopic images. Our results further suggested that the apoptotic role of DHA involves caspase-3 activation. Our results revealed a novel function of DHA in the restoration of healthy balance between VSMC proliferation and apoptosis, demonstrating a potential strategy for the treatment of HG fluctuations induced diabetic atherosclerosis complications. Future studies would focus on further investigation of evaluating the molecular signalling mechanism involved in these apoptotic pathways.

## Figures and Tables

**Fig. (1):**
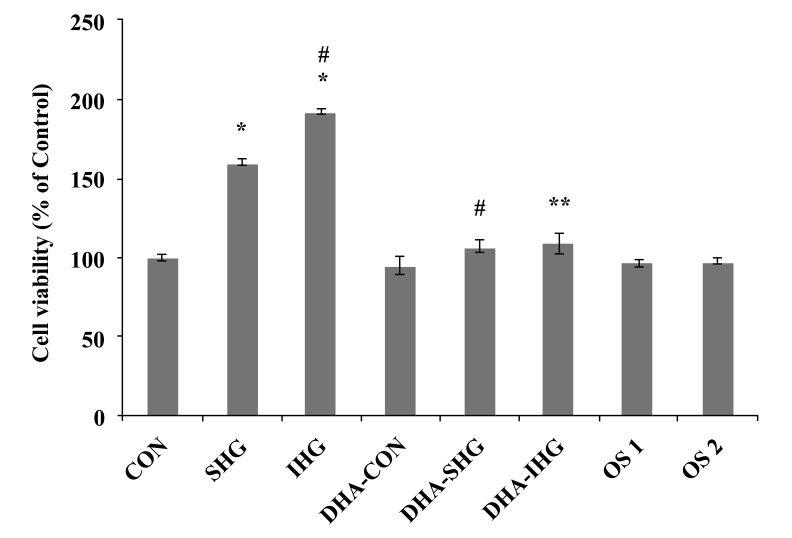
Effect of DHA on the proliferation of VSMCs induced by IHG. Quiescent cultures of VSMC were pretreated with DHA and exposed to constant or intermittent high glucose level for 72 hrs. After treatment, cell survival was examined using the MTT reduction assay. Cell viability was calculated as the percentage of the control group. The data represent mean ± SEM of 3 individual experiments. *P < 0.001 versus Con, # *P* < 0.001 versus SHG, ** *P* < 0.001 versus IHG.

**Fig. (2):**
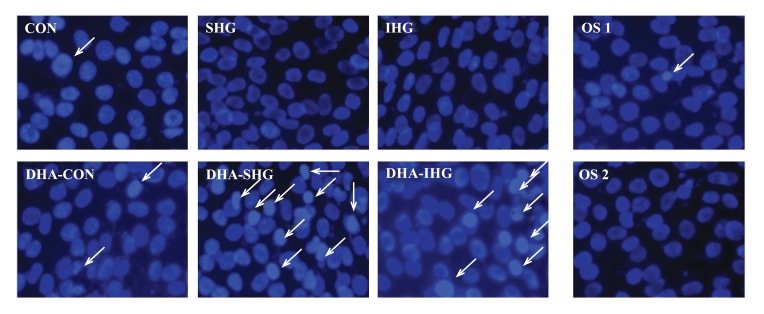
Effects of DHA on the nuclear morphology of HG-treated VSM cells stained with Hoechst 33258. The Con group showed cells with uniformly dispersed chromatin and weak fluorescence signals. DHA-treated SHG and IHG group demonstrated hypercondensed chromatin with increased number of apoptotic cells.

**Fig. (4):**
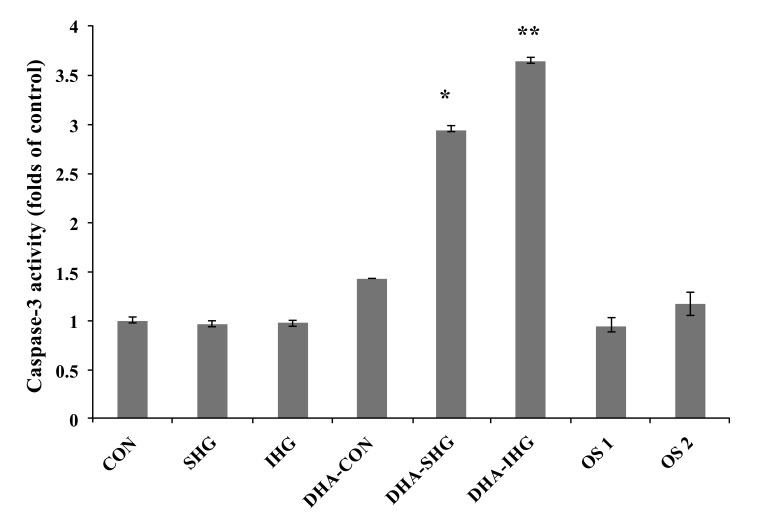
Effects of DHA pre-treatment on caspase-3 activity in HG-treated VSMC. The VSMCs seeded in a 96-well plate were treated as described in the Methods section. The caspase 3 activity was examined by measuring the fluorescence of the N-Ac-DEVD-N’-MC-R110 substrate in the plate reader with an excitation wavelength at 485 nm and an emission wavelength at 535 nm. Data represents mean ± SD of 3 individual experiments with * *P* < 0.001 versus SHG, ** *P* < 0.001 versus IHG.

## LIST OF ABBREVIATIONS

DHA-: = Docosahexaenoic Acid
HG-: = High Glucose
IHG-: = Intermittent High Glucose
MTT: = -3-(4, 5-dimethyldiazol-2-yl)-2, 5-diphenyltetrazolium bromide.
PUFA -: = Polyunsaturated Fatty Acid(s)
SHG-: = Stable High Glucose
VSMC: = Vascular Smooth Muscle Cells
